# Prediction of Auditory and Visual P300 Brain-Computer Interface Aptitude

**DOI:** 10.1371/journal.pone.0053513

**Published:** 2013-02-14

**Authors:** Sebastian Halder, Eva Maria Hammer, Sonja Claudia Kleih, Martin Bogdan, Wolfgang Rosenstiel, Niels Birbaumer, Andrea Kübler

**Affiliations:** 1 Institute of Psychology, University of Würzburg, Würzburg, Germany; 2 Institute of Medical Psychology and Behavioral Neurobiology, University of Tübingen, Tübingen, Germany; 3 Wilhelm-Schickard Institute for Computer Science, University of Tübingen, Tübingen, Germany; 4 Ospedale San Camillo, Istituto di Ricovero e Cura a Carattere Scientifico, Venezia, Italy; 5 Computer Engineering, University of Leipzig, Leipzig, Germany; Okayama University, Japan

## Abstract

**Objective:**

Brain-computer interfaces (BCIs) provide a non-muscular communication channel for patients with late-stage motoneuron disease (e.g., amyotrophic lateral sclerosis (ALS)) or otherwise motor impaired people and are also used for motor rehabilitation in chronic stroke. Differences in the ability to use a BCI vary from person to person and from session to session. A reliable predictor of aptitude would allow for the selection of suitable BCI paradigms. For this reason, we investigated whether P300 BCI aptitude could be predicted from a short experiment with a standard auditory oddball.

**Methods:**

Forty healthy participants performed an electroencephalography (EEG) based visual and auditory P300-BCI spelling task in a single session. In addition, prior to each session an auditory oddball was presented. Features extracted from the auditory oddball were analyzed with respect to predictive power for BCI aptitude.

**Results:**

Correlation between auditory oddball response and P300 BCI accuracy revealed a strong relationship between accuracy and N2 amplitude and the amplitude of a late ERP component between 400 and 600 ms. Interestingly, the P3 amplitude of the auditory oddball response was not correlated with accuracy.

**Conclusions:**

Event-related potentials recorded during a standard auditory oddball session moderately predict aptitude in an audiory and highly in a visual P300 BCI. The predictor will allow for faster paradigm selection.

**Significance:**

Our method will reduce strain on patients because unsuccessful training may be avoided, provided the results can be generalized to the patient population.

## Introduction

Brain injuries or neurological diseases (e.g. amyotrophic lateral sclerosis, ALS) can lead to complete motor paralysis. Depending on the degree of impairment caused by the injury or the progression of the disease communication can become very difficult and even impossible. This loss of communicative abilities can be overcome with interfaces that bypass the need for muscular control and detect the user's intentions directly from signals recorded from the brain. These brain-computer interfaces (BCIs) are currently not only used for communication but also for restoration of motor control [1], [2], [3], [4].

In all variations of BCIs a control signal must be recorded from the user's brain. Most often this signal is extracted from the electroencephalogram (EEG). The EEG has the advantage of reliable, economic and portable recording devices. Disadvantages are the strong attenuation of the neural signals by the skull and skin and long preparation times if many EEG electrodes (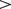
32) are applied. Despite these disadvantages, up to now for working with severely paralyzed patients EEG, remains the most practical method.

One commonly used control signal in the EEG is a positive deflection designated as P300. It is elicited by unexpected stimuli with variations in latency between 250 and 700 ms on central to parietal locations [5]. BCIs using the P300 as control signal have been evaluated extensively with ALS patients in several independent studies [6], [7], [8], [9], [10], [11]. Typically, the user focuses on an element in a matrix with for example 6×6 symbols. In one run, rows and columns of the matrix flash randomly. The user focuses on the desired item (target) and a P300 is elicited whenever the target row or column are flashing. This experimental design has not only been used for spelling but also for control of internet browsers [12], painting [13], control of standard assistive devices [11] and wheelchair control [14]. A negative side effect of using row and column based flashing patterns is that errors may occur by selecting a wrong letter in either the correct column or row which may be compensated by using e.g. a checkerboard based flashing pattern [15] or online error correction [16].

When considering the applicability of a BCI to enhance or restore communication it is important to differentiate between various states of impairment. Particularly, the distinction between the locked-in state (LIS) and the complete locked-in state (CLIS) is important when considering the application of a P300 BCI [17]. The ability of the user to control gaze, which is lost in late-stage ALS and by definition in CLIS, is required by most common implementations of this BCI, some approaches work around this [18], [19], [20]. The limitation of visual P300 BCIs to patients with functional gaze control is addressed by P300 BCI implementations that use auditory or tactile instead of visual stimulation. Control of a visual P300 BCI is possible without direct fixation of the target but this substantially decreases accuracies [21]. Addtionally, it may be possible that patients have uncontrollable eye drifts preventing them from fixating any point in space. It is possible to transfer the P300 speller to the auditory domain by using defined auditory stimuli instead of flashing for each row and column. These auditory stimuli can be either words or sounds [22], [23], [24]. Another approach is to reduce the number of possible choices to make selection faster [8]. Load can be reduced by approaches using very distinct auditory stimuli instead of words [25], [26]. By stimulation from different spatial directions the number of classes and thus communication speed can be increased [27], [28]. [22] pointed out differences between the P300 elicited by the visual and the auditory P300 BCI. The latencies of the P300 elicited by the auditory BCI were increased compared to the latencies elicited by the visual P300 BCI. Peak amplitudes were identical but the maximum peak of the auditory P300 BCI occured on posterior instead of central electrodes.

Considering that BCIs are currently primarily intended for patients who are diagnosed with severe diseases that not only lead to motor impairment but also to reduced attention span it would be advantageous to be able to quickly choose a suitable BCI and training strategy that best fits the patients needs [3]. For this reason reliable predictors of aptitude with a particular BCI are necessary. Ideally, data for prediction should be collected quickly and without the active participation of the user. This is particularly important if the user cannot respond or communicate because of CLIS. Such a predictor has been presented for sensorimotor rhythm (SMR) based BCIs [29]. The amplitude of the SMR peak while the user is resting was used as a neurophysiological predictor which correlated highly with BCI aptitude in a sample of healthy individuals (Pearson's 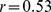). It is important to note that this predictor was obtained when the participant was not performing the motor imagery task, and thus the predictor was independent of any mental strategy applied for later cursor control. Therefore, and most importantly the data necessary for SMR-BCI aptitude prediction can be obtained from non-responsive patients. Furthermore, psychological variables such as the ability to concentrate and visuo-motor coordination also predicted SMR BCI aptitude [30]. Likewise, a high correlation has been shown between BCI aptitude and the hemodynamic response in the dorsolateral prefrontal cortex, an area known to be involved in task monitoring [31].

Studies with users of BCIs controlled by regulation of slow cortical potentials (SCP) demonstrated the predictive power of accuracy in early sessions for later accuracy [32]. More specifically, the number of sessions needed to achieve significant cursor control correlated moderately with the number of sessions required to achieve criterion level control (above 70%, [33]). The implicit learning capacity also appeared to influence the ability to use SCP BCIs [34]. Concerning P300 BCIs, it has recently been shown that motivation may impact accuracy achieved in a subsequent BCI session [35]. Due to a ceiling effect in BCI accuracy (an average accuracy of 99% was achieved by N = 33 participants) the effects of motivation on online accuracy could not be studied due to a lack of variance, but P300 amplitude was reduced in the least as compared to the highest motivated participants. [36] deomonstrated that heart rate variability (HRV) recorded during a 10 minutes rest period without any task and EEG recording moderately predicted later P300 BCI performance in healthy subjects. HRV is a correlate of cortical inhibition and more globally, of self-regulatory capacity.

Thus, to date no strong predictor of P300-BCI aptitude is available. In the current study we propose to predict auditory AND visual P300-BCI aptitude from a single auditory standard oddball measurement. An auditory as opposed to a visual oddball was used because it can be applied to patients without gaze control. The type of auditory oddball used (one rare target tone, one frequent non-target tone with different physical properties) is by design in fact more similar to the visual P300 BCI experiment in which the user attends a single element of the matrix which flashes infrequently and is dark most of the time than to the auditory P300 BCI experiment. Thus, there is also an infrequent target and a frequent target distinguished by physical properties. In case of the auditory P300 BCI the target and non-targets are distinguished by attention to the semantic meaning of the target word whereas the physical properties of targets and non-targets are more similar. The goal of this study was to determine how strongly the amplitude and waveform of the P300 elicited by the auditory standard oddball would correlate with subsequent BCI aptitude. We recorded an auditory standard oddball session from each subject, followed by a single visual P300 BCI and auditory P300 BCI session. Subsequently the amplitude of auditory standard oddball response was correlated with BCI accuracy.

## Methods

### Participants

Forty healthy participants (21 male, 19 female, mean age 25.8 years, SD 8.46 years, range 17–58) took part in the study which was approved by the Ethical Review Board of the Medical Faculty, University of Tübingen. Each participant was informed about the purpose of the study and signed informed consent prior to the experimental session.

### Experimental design

**Figure 1:**
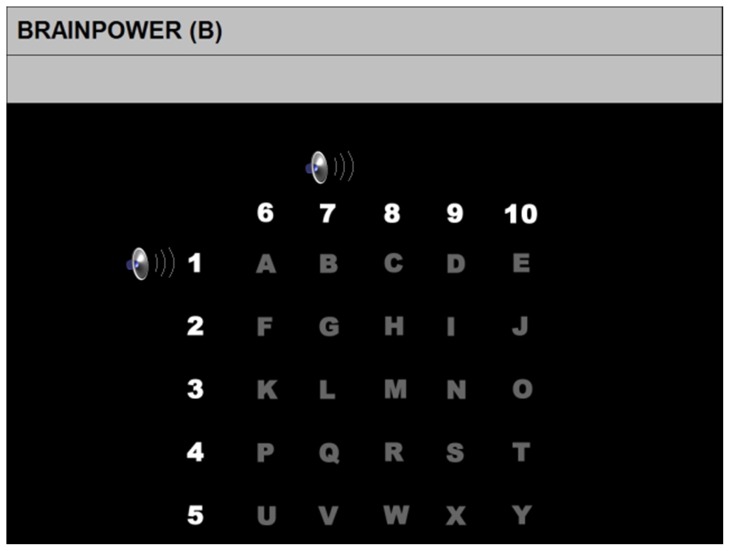
Visual support matrix displayed on a computer screen to the participant during the auditory P300-BCI experiment [22]. The matrix was identical to the visual P300 BCI matrix. The speakers displayed at the top left corner of the matrix indicate the auditory presentation of numbers and were not displayed during the actual experiment.

**Figure 2:**
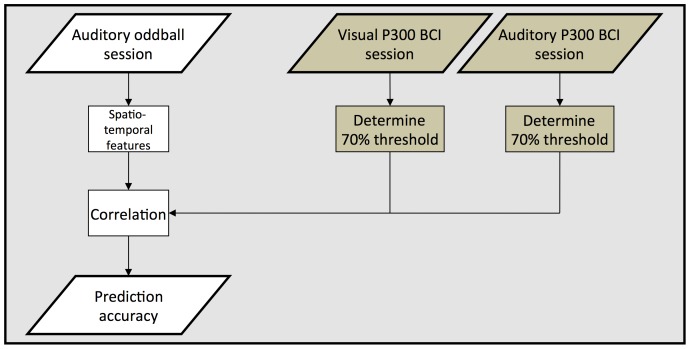
Every participant was presented with an auditory oddball, and performed a visual and auditory P300 BCI session. For both visual and auditory P300 BCI online feedback was provided. Performance was reevaluated offline by calculating the number of repetitions per stimulus the user needed to reach 70% accuracy. This performance measure was correlated with features extracted from the auditory oddball session to assess whether it can be used to predict BCI aptitude.

The participants were seated in a comfortable chair approximately 1 m away from a digital computer screen (43 cm diameter). Conventional headphones were used to present the auditory stimuli. Participants were cued about the beginning and the end of a run (see below) by the German word “Warte . . .” (engl.: “Waiting . . .”) and “Zeit abgelaufen!” (engl.: “Time out!”). The screen was blank while the auditory oddball was presented, displayed a visual support matrix (see Figure 1) during the auditory or flashing rows and columns during the visual P300 BCI experiment. Figure 2 depicts an overview of all parts of the experiment.

### Auditory oddball

Auditory stimuli were standard tones (duration 160 ms; overlapping 517 Hz, 646 Hz and 775 Hz tones) and deviants (the oddball; duration 160 ms; a 517 Hz tone) with a ratio of 4:1. The tones were two distinct harmonic oscillations each consisting of three frequencies instead of pure sine tones. Such complex tones have been shown to elicit a larger P300 [37]. Stimuli were presented to the user in sequences of five tones, namely four standards and a deviant. Each run comprised 20 sequences, wherein the order of stimuli was randomized. Each run was repeated three times resulting in a total of 60 deviant and 240 standard tones. A group of three runs will be referred to as a session. Participants were instructed to count the deviants and after each run were asked for the number of deviants. The inter-stimulus interval was set to 800 ms. Thus, one run lasted 96 s. In between runs participants were asked if they were ready to continue, and if yes the next run commenced. Altogether the session lasted 288 s (4 min 48 s). The data (see below) of this experiment was used to predict the aptitude of the participants in the following two experiments.

### Visual P300 BCI

During the visual P300 BCI experiment participants were presented with a 5×5 matrix containing the letters of the latin alphabet excluding the letter Z to make it compatible to the auditory P300 BCI experiment [22]. One sequence comprised 10 flashes (one for each row and column) of 80 ms duration followed by a 160 ms inter-flash interval. To select a letter 15 sequences were required followed by 2.4 s breaks in which the signal was classified and the selected letter was presented to the participant. In total, a single letter selection required 38.4 s. This relativly long letter selection time is caused by the use of 15 sequences per selection which were needed to make the visual P300 BCI comparable to the auditory paradigm. With differing sequence numbers some of the analysis methods used would not have been possible. Offline performance was recalculated for each participant to obtain a measure of individualized performance (see Section “Offline performance measure”). One run comprised selection of five letters and participants performed 6 runs (30 letters). In the first two runs participants spelled the words BRAIN and POWER and no feedback of results was provided; this data was used to train the classifier and participants were grouped into high aptitude and low aptitude users according to the accuracy achieved in runs 3 to 6 with feedback (alternating the words BRAIN and POWER). Accuracy was measured as percentage of correctly selected letters.

### Auditory P300 BCI

The auditory matrix was identical to the visual P300 BCI described above (see Figure 1). Instead of flashes, auditory stimuli were presented to the participant. Each row and column was coded by the number presented at the top of the columns and left to the rows (see Figure 1). The auditory stimuli were the corresponding pre-recorded, spoken number. The 5×5 matrix with corresponding numbers was displayed on the screen throughout auditory stimulus presentation, but no flashing of rows and columns occurred. The stimulus presentation time was 450 ms as in [22] but the inter stimulus interval (ISI) was increased from 175 to 550 ms because preliminary results indicated this would increase accuracy of spelling and higher ISIs would be needed by patients [38], [39], [40]. To reduce cognitive load, rows and columns were sequentially selected: within a sequence first the rows and then the columns were presented to the participant. As in the visual P300 BCI experiment each letter selection consisted of 15 sequences with intervals of 2.4 s between each selection. Thus, time needed for selection of one letter were 150 s. Again the words BRAIN and POWER were presented three times each with the first ten letters being used for classifier training without presenting feedback to the participant. Subsequently, selected letters (20) were presented to the subject. Again, accuracy was defined as the percentage of correctly selected letters.

It is not possible to operate the auditory P300 BCI used in this study (which is based on spoken numbers as stimuli) with speeds identical to those of the visual P300 BCI. It would also not have been realistic to operate the visual P300 BCI with ISIs identical to those of the auditory P300 BCI. Thus, using an identical number of sequences appeared to be the best way to allow for a certain measure of comparability. The high number of sequences also ensured that the point at which 70% accuracy were achieved could be calculated for most participants.

### Data acquisition

All aspects of data collection were controlled by the BCI2000 software system [41]. The EEG was recorded with Ag/AgCl elctrodes in a 128-channel cap (Easycap GmbH) of which 63 EEG and 4 electrooculography (EOG) channels were used. The electrodes were located at the following positions with the channel number in parentheses: FP1(1), Fpz(2), FP2(3), F7(4), F3(5), Fz(6), F4(7), F8(8), FT9(9), FT7(10), FC5(11), FC3(12), FC1(13), FCz(14), FC2(15), FC4(16), FC6(17), FT8(18), FT10(19), T7(20), C5(21), C3(22), C1(23), Cz(24), C2(25), C4(26), C6(27), T8(28), TP9(29), TP7(30), CP5(31), CP3(32), CP1(33), CPz(34), CP2(35), CP4(36), CP6(37), TP8(38), TP10(39), P9(40), P7(41), P5(42), P3(43), P1(44), Pz(45), P2(46), P4(47), P6(48), P8(49), P10(50), PO7(51), P05(52), P01(53), POz(54), P06(55), P02(56), PO8(57), O1(58), Oz(59), O2(60), O9(61), Iz(62), O10(63). The locations of the electrodes were based on the modified 10–20 system of the American Electroencephalographic Society [42].

Each channel was referenced to the tip of the nose and grounded to a position between Fz and Fpz. Eye movement and blinks were recorded using two vertical EOG channels with electrodes placed above and below the right eye (superior and inferior orbital fossa), and two horizontal EOG channels with electrodes placed at the outer canthi of the eyes. Impedances were kept below 5 k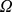. The EEG was recorded using a BrainAmp DC Amplifier (Brainproducts GmbH), notch-filtered at 50 Hz and sampled at 500 Hz. The resolution was set to 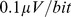. With this setting the amplifier samples with a rate of 5 kHz with an internal low-pass at 1 kHz; such oversampling prevents aliasing. The decimation to the recording frequency (in this case 500 Hz) is performed within the Brainvision Recorder software provided by the amplifier manufacturer. No additional filtering was applied to the online recording. Data processing, storage and online display were performed on a conventional PC (Intel Core 2 Quad Q6600 2.40 Ghz, 4 GB RAM, Microsoft Windows XP Professional SP2 32-bit).

### Offline processing

The data acquired during presentation of the auditory oddball were high-pass filtered at 0.5 Hz and then low-pass filtered at 20 Hz using two-way least-squares finite impulse response (FIR) filtering with a function from the EEGLAB toolbox [43].

The blind source separation (BSS) algorithm AMUSE was used to isolate and remove ocular artifacts [44], [45]. It has been shown to be superior to higher order statistics BSS algorithms in both speed and separation performance [46]. For offline analysis the nose reference was replaced with a common average reference (CAR). After segmenting the data into individual epochs (0–800 ms), they were baseline corrected by subtracting from every epoch the mean amplitudes in the −100 to 0 ms pre-stimulus interval.

### Classification

We used stepwise linear discriminant analysis (SWLDA) for online and offline classification. This method, an extension of Fisher's linear discriminant analysis, is an established classification method for visual and auditory P300 BCIs [6], [22], [47], [48], [49]. The spatiotemporal features (the channel by sample matrix) of each trial were smoothed with a moving average filter, with a width of 25 samples, and then decimated by a factor of 25 prior to feature selection and classification. The algorithm starts with adding the most significant feature to the model (at least 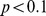, otherwise the model generation fails). It then iterates across the remaining features in order of their significance. Each time a feature proves significant it is added to the model. Addtionally, a backward stepwise regression is performed to remove features above the predefined significance threshold (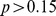). This continues until a maximum of 60 iterations have been performed or no more features meet the significance threshold for inclusion (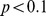).

For online classification of P300 signals the model was applied to the trials of each row and column separately. The row and the column which yielded the maximum score after model application were selected by the classifier. This meant that for classification of the P300 responses in the BCI no bias term was required.

### Offline performance measure

Due to a ceiling effect of online visual P300 BCI performance (100% accuracy for 28 of 40 participants) the data was reclassified offline using all six runs of each participant in a leave-one-run-out cross validation loop. To prevent the reoccurance of ceiling effects not the overall accuracy after 15 stimulus repetitions was used but the number of sequences needed to achieve 70% accuracy (the criterion level of control). The number of repetitions was linearly interpolated to non-integer numbers.

### Information transfer rate

Information transfer rate in BCIs is commonly assessed using bits/min as defined by [50]. In P300 BCIs on the basis of flashing matrices this formula is not valid due to unevenly distributed error probabilities [51]. The probability of selecting a neighbor row or column is higher than that for selecting more distant rows and columns. Therefore, it has been suggested to use mutual information to calculate the information transfer rate [52], [53]. All error probabilities were calculated based on the selections of the 40 participants of this study.

### Correlations

For comparisons the group was split at the median of sequences needed to achieve 70% accuracy and rank correlations were performed according to [54].

## Results

### BCI performance

Mean accuracy was 94.5% (SD 14.7, range 35–100, N = 40) for the visual and 62.9% (SD 38, range 0–100, N = 38) for the auditory P300-BCI. Two participants had to be excluded from the auditory P300 BCI study due to technical problems during the online recording. One of these two participants could be reclassified offline. Therefore the aptitude prediction for the auditory P300 BCI is based on N = 39 participants. T-test for dependent samples proved significant (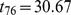, p
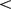
0.001) indicating that participants performed worse with auditory presentation of stimuli.

**Figure 3:**
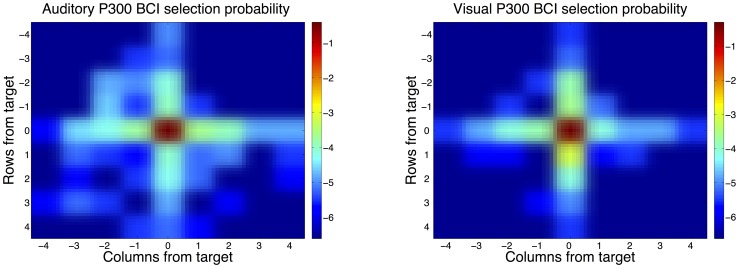
Probability of selecting the target matrix element (center) or a matrix element around the target is color coded on a logarithmic scale. The x-axis shows how many columns to the left (negative) or to the right (positive) an error occurs. Correspondingly, the y-axis shows the probabilities for errors for rows above or below the target. Both in the visual P300 BCI (left) and the auditory P300 BCI (right) errors occur with a much higher probability on the same row or column as the target. This unequal distribution of the error probability was the motivation for applying mutual information to measure bitrate.

An ITR of 4.7 bits/min was achieved for the visual P300-BCI with 15 sequences and of 18.4 bits/min for three sequences offline. With 0.8 bits/min the performance of the auditory P300-BCI with 15 sequences was lower. The spatial distribution of errors is visualized in Figure 3.

**Figure 4:**
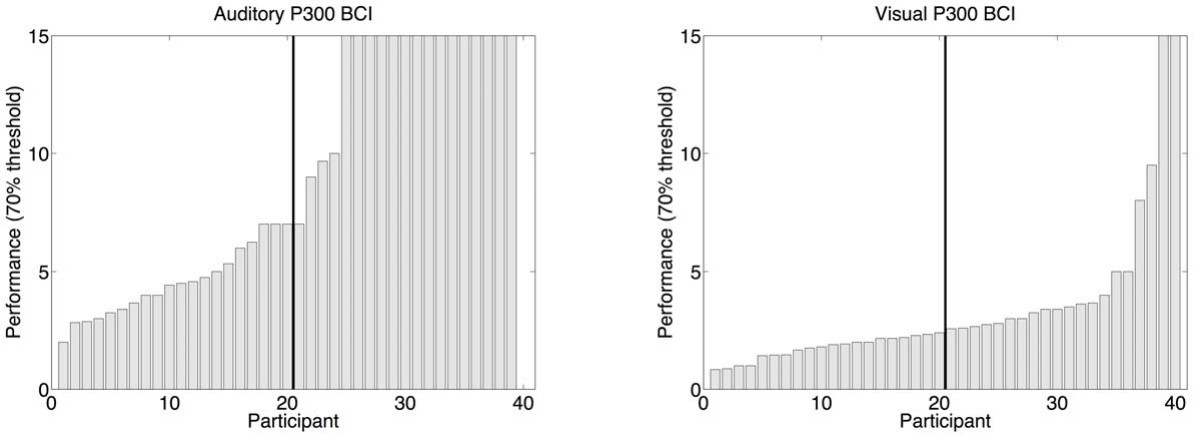
Performance distributions for auditory P300 BCI (left) and visual P300 BCI (right). The median is indicated by a vertical black line.

**Figure 5:**
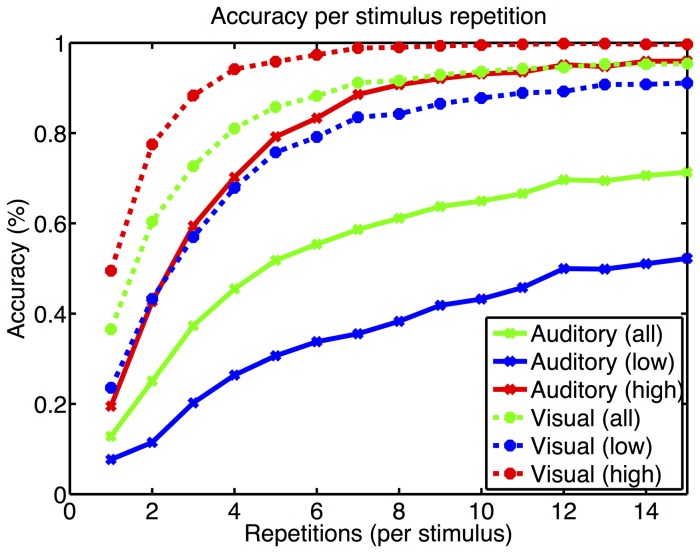
The letter selection accuracy is plotted as a function of the number of stimulus repetitions, i.e. flashes or spoken row/column numbers. Data from all 63 EEG channels and a time window of 800 ms was available for the classifier. It can be seen that in the auditory modality only high aptitude users achieve an error rate below 20% comparable to the visual modality for all users. Dashed lines: visual P300 BCI; Continuous lines: auditory P300 BCI.

For each experiment we used median split to form groups of high and low aptitude users based on the performance calculated offline, i.e. the number of stimulus repetitions needed to reach 70% accuracy. This measure was calculated for each participant and experiment (visual or auditory P300 BCI) separately. Auditory and visual P300 online and offline BCI performance correlated moderately (online 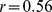, 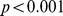; offline 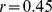, 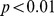). On average 3.41 repetitions (10 seconds, 6 selections/min) were needed to achieve 70% with the visual P300 BCI whereas 9.01 (92.5 seconds, 0.64 selections/min) were needed with the auditory P300 BCI. Low aptitude visual P300 BCI users needed 5.10 repetitions (14.6 seconds, 4.1 selections/min) and auditory 12.80 (130.4 seconds, 0.46 selections/min). High aptitude visual P300 BCI users needed 1.73 sequences (6.5 seconds, 9.1 selections/min) and auditory 4.11 (43.5 seconds, 1.4 selections/min). The distribution of performance across particpants is shown in Figure 4. The mean offline accuracy achieved as a function of stimulus repetitions is shown in Figure 5.

**Figure 6:**
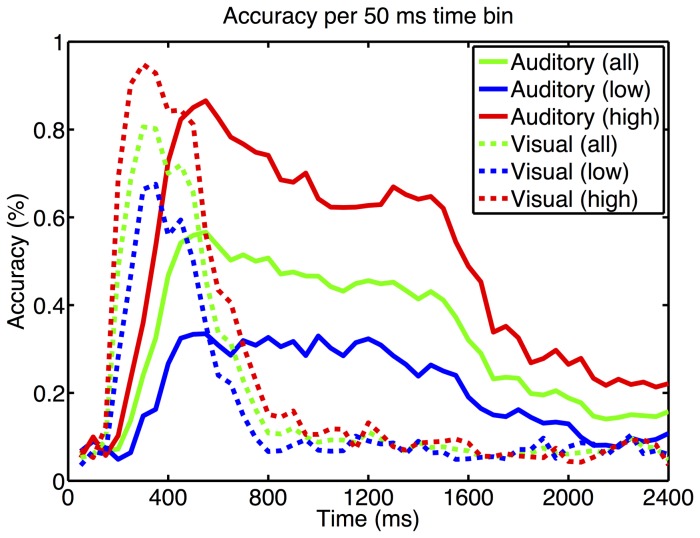
The letter selection accuracy is plotted as a function of time. The data was split into non-overlapping 50 ms time bins that were used to train and test the classifier. Data from all 63 EEG channels was available for the classifier. For the visual P300 BCI the highest accuracy occurs in the expected P300 time window. In the auditory BCI neither the high nor the low aptitude users achieve an accuracy as high as in the visual P300 BCI. Dashed lines: visual P300 BCI; continuous lines: auditory P300 BCI.

Besides determining the dependency of accuracy on the number of stimulus repetitions we determined which time segments provide the best classification accuracy (see Figure 6). The data was segmented into windows of 50 ms length that were classified individually. With the visual P300 BCI high aptitude user achieved best performance (94.83% accuracy) around 300 ms. Low aptitude users achieved the maximum performance (67.53% accuracy) in the window around 350 ms. With the auditory P300 BCI performance peaked around 550 ms for both groups. High aptitude users had an accuracy of 86.55% and low aptitude users of 33.48%. The visual P300 BCI accuracy dropped to chance level at around 950 ms whereas the auditory P300 BCI data could be classified well above chance until about 1700 ms.

### Event-related potentials

**Figure 7:**
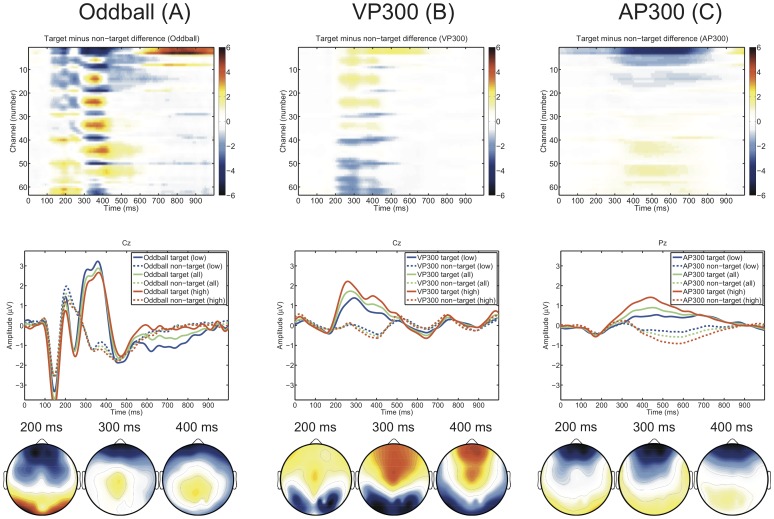
Responses to auditory oddball (A), visual P300 BCI (B) and auditory P300 BCI (C) are shown from left to right. Top row: average amplitude of the full spatio-temporal feature matrix of the target non-target difference for each experiment. Middle row: time course at Cz (auditory oddball and visual P300 BCI) and Pz (auditory P300 BCI) of the averaged ERP for targets (continuous lines) and non-targets (dashed lines). Bottom row: topographic distribution of the target non-target difference at 200, 300 and 400 ms. For the auditory oddball, subjects were split in high and low aptitude users at the median of the mean performance in the auditory and visual P300 BCI.

Figure 7 shows a comparison of the ERPs elicited by the auditory oddball (A), the visual P300 BCI (B) and the auditory P300 BCI (C). The top row shows the color-coded differences in amplitude between target and non-target responses. The auditory oddball elicited the strongest responses with differences between targets and non-targets beginning at 100 ms. In response to the auditory oddball a clear N1-P2-complex can be seen which is not present in the auditory P300 BCI. The latency of the P300 event-related potential was much higher and the amplitude lower in the auditory P300 BCI. The mean P3 amplitude and latency (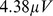 at 363 ms) did not differ between high (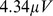 at 385 ms) and low aptitude users (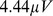 at 346 ms; amplitudes 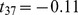, 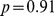; and latency: 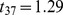, 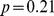).

In the visual P300 BCI the mean amplitude but not latency (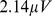 at 309 ms) of high (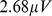 at 297 ms) and of low (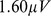 at 319 ms) aptitude users differed significantly (amplitude: 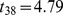, 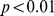; latency: 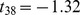, 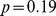).

The latencies of the ERPs elicited by the auditory P300 BCI task have the lowest amplitudes and longest latencies of all investigated tasks (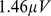 at 508 ms). Again the amplitudes of the high aptitude users (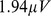) differ significantly from those of the low aptitude users (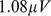; 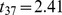, 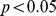). Again, latencies do not differ between high (516 ms) and low aptitude users (502 ms; 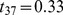, 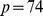).

Both the auditory oddball and the auditory P300 BCI elicited an anterior-negative, posterior-positive distribution of amplitude differences whereas the visual P300 BCI elicited an anterior-positive, posterior-negative distribution.

### Correlation of auditory oddball response with BCI performance

**Figure 8:**
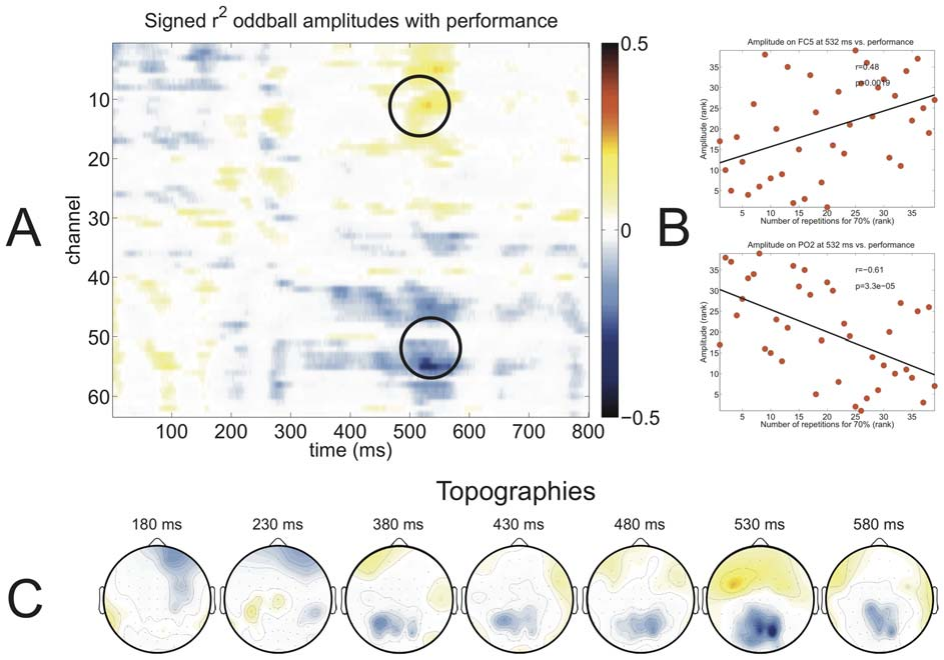
Signed 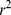 values between auditory oddball amplitudes of all time points and channels with *auditory* P300 BCI performance (defined as the number of sequences needed to reach 70% accuracy) are shown in red for positive correlations and in blue for negative correlations (A). Two elements from the matrix were selected for visualization using scatter plots (B) showing a correlation of 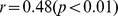 on electrode FC5 and a correlation of 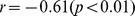 on electrode PO2. Topographic distributions of the signed 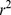 values are shown at the bottom (C). Note that due to the use of “number of sequences needed to reach 70% accuracy” as performance measure positive correlations indicate a decrease in performance with increasing amplitude, whereas negative correlations indicate an increase of performance with decreasing amplitude.

**Figure 9:**
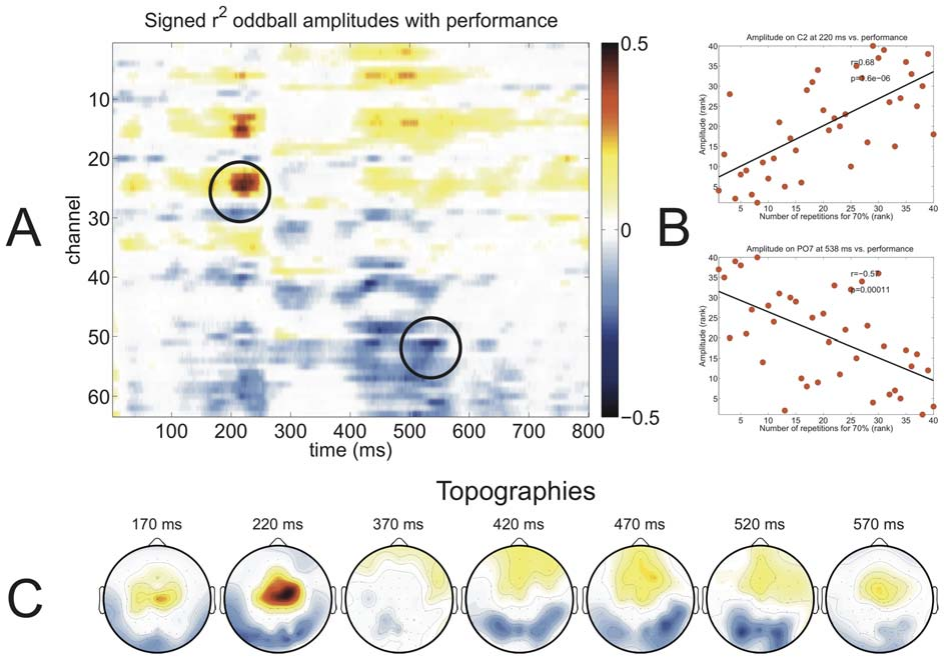
Signed 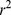 values between auditory oddball amplitudes of all time points and channels with *visual* P300 BCI performance (defined as the number of sequences needed to reach 70% accuracy) are shown in red for positive correlations and in blue for negative correlations (A). Two elements from the matrix were selected for visualization using scatter plots (B) showing a correlation between performance and amplitude of 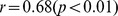 on electrode C2 and of 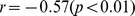 on electrode PO7. Topographic distributions of the signed 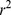 values are shown at the bottom (C). Note that due to the use of “number of sequences needed to reach 70% accuracy” as performance measure positive correlations indicate a decrease in performance with increasing amplitude, whereas negative correlations indicate an increase of performance with decreasing amplitude.

The main goal of this experiment was to determine the predictive power of the auditory oddball on visual and auditory P300 BCI performance. Figures 8 and 9 depict the correlation between the auditory oddball ERP amplitude with auditory (Figure 8) and visual (Figure 9) P300 BCI performance. As noted in the captions of the figures, lower values in the performance metric indicate better performance. Therefore, a blue coloring indicates that higher auditory oddball amplitudes imply better performance. For both the auditory and the visual P300 BCI an increased anterior-negative, posterior-positive amplitude of auditory oddball responses between 400 and 600 ms coincided with high performance (Figure 8 (A) and 9 (A)). In case of the visual P300 BCI there was also a high correlation between oddball amplitudes and performance in the 200 and 250 ms time window (Figure 9 (A)). For both BCI paradigms we could not observe the strongest correlation in the time window of 250 and 400 ms in which the P300 occurred. The elements of the matrix with the strongest positive and negative correlation in Figures 8 (A) and 9 (A) are visualized in Figures 8 (B) and 9 (B). In the auditory BCI both the highest positive (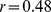, 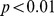) and highest negative correlation (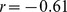, 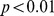) occured at 532 ms on frontal (FC5) and posterior (PO2) electrodes, respectively. In the visual BCI the strongest positive correlation occurred in the 200 to 250 ms time window at electrode C2 at 220 ms (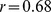, 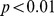). As with the auditory BCI the strongest negative correlation was found on a posterior electrode (PO7) at 538 ms (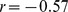,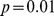). The auditory oddball amplitude exhibited correlations with performance of both paradigms resembling the morphology of an anterior-negative, posterior-positive wave between 400–600 ms which can be seen in the topographies of Figures 8 (C) and 9 (C). Additionally, 9 (C) depicts the strong positive correlation at central electrodes at 220 ms. Based on these observations, i.e. that an early potential in the N2 time range and a late potential between 400 and 600 ms correlated with BCI performance whereas the P300 elicited by the auditory oddball did not, we performed an individual peak amplitude and latency detection for each participant and correlated these values with performance (see Table 1). For each participant the latency of the P300 was determined first (maximum between 250 and 700 ms at Cz), then the N200 was defined as the minimal amplitude before the P300 (also at Cz) and finally the late potential was defined as the maximum amplitude after the P300 (at POz). Average amplitudes of all three ERP components can be found in Table 1. The amplitude of the N2 component correlated significantly with auditory and visual P300 BCI performance, the amplitude of the P300 correlated with neither and the amplitude of the late positive component on POz correlated negatively only with visual P300 BCI performance (see Table 1, left half). Except the negative correlation between latency of the P300 component and auditory P300 BCI performance (p = 0.04) none of the correlations between latency and performance were significant (see Table 1, right half).

**Table 1:** Amplitudes, latencies and correlations thereof with BCI performance shown for N200 (minimal amplitude before latency of P300 on Cz), P300 (maximum between 250 and 700 ms on Cz) and late ERP component (maximum after P300 latency on POz).

	Amplitude (*µ*V)	R auditory	R visual	Latency (ms)	R auditory	R visual
N200 (Cz)	−3.25 (SD 2.25)	**0.37 (p = 0.02)**	**0.47 (p<0.01)**	229.05 (SD 42.57)	−0.22 (p = 0.18)	0.04 (p = 0.81)
P300 (Cz)	4.99 (SD 2.66)	0.04 (p = 0.81)	−0.05 (p = 0.75)	378.00 (SD 89.00)	**−0.32 (p<0.05)**	0.07 (p = 0.65)
Late ERP (POz)	3.61 (SD 2.10)	−0.26 (p = 0.12)	**−0.46 (p<0.01)**	548.65 (SD 168.55)	0.07 (p = 0.66)	0.19 (p = 0.25)

## Discussion

We presented P300 BCI performance data from healthy participants using a visual and an auditory P300 BCI. A standard oddball measurement lasting less than five minutes was used to predict the performance of the participants in the BCI application. The largest differences between high and low aptitude P300 BCI users in the response to the standard oddball were the amplitude of the N2 response on Cz and a late postive potential at POz. Correlation between N2 and performance was 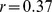 (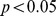) for the auditory P300 BCI and 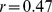 (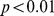) for the visual P300 BCI. Correlation between the late potiential and performance was only significant for the visual P300 BCI (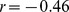; 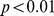).

### BCI Performance

Visual P300 BCI online accuracy of the 40 participants was on average 94.5%. This level of accuracy is what can be expected with healthy participants when using the standard visual P300 BCI and is therefore comparable to that achieved in other studies [15], [22], [35], [55]. Comparisons between experimental designs are difficult due to different time intervals, number of sequences and channel sets. In any case, the ceiling effect is a common phenomenon. It might therefore be recommendable to use three repetitions for visual P300 BCI letter selections in studies that analyze influences on or predictability of performance as we found this value to lead to an average accuracy of 70% in our sample and normal distribution of correct response rate. This accuracy is still sufficiently high for comprehensible spelling while effectively removing the ceiling effect.

In contrast, with 62.9%, the mean accuracy with the auditory P300 BCI was considerably lower. Based on previous studies which indicated that a longer ISI would increase accuracy we used an ISI of 550 ms instead of 175 ms as was used in initial study with the auditory P300 BCI designed by [22]. However, online accuracy levels were comparable to the 65% achieved by [22] and thus, were not improved by the longer ISI. Due to the decreased selection speed, information transfer rates were much lower reaching only 0.8 bits/min (compared to 1.54 bits/min in [22], albeit this was calculated using the formula by [50]). Nonetheless, our data indicate that increases in information transfer rate may easily be achieved. For instance, accuracies of 60% are already achieved with nine sequences in the auditory P300 BCI. Using only nine sequences the information transfer rate would increase to 1.2 bits/min. Alternatively the ISI could be decreased to the values used by [22] which would also increase the information transfer rate to 1.2 bits/min. Compared to the visual P300 BCI the development of the auditory P300 BCI is in a fairly early stage. At the time the data of this study was collected (2008) no other functioning auditory P300 speller system existed. Online ERP based auditory BCIs with a reduced number of selections [8], [25] were available but we preferred a system comparable to the visual P300 BCI. Currently a BCI system using spatially distributed auditory stimuli for target selection appears to be the most promising path of future development [27].

### BCI ERPs

As in the study by [22] we found higher latencies in response to the target stimuli of the auditory P300 BCI paradigm than of the visual P300 BCI. This is an effect that was also observed in non-BCI related ERP studies [56]. As noted by [22] this may be due to a general increase in the synaptic delays in the auditory cortex as compared to the visual [57]. On the other hand, it has been shown that general P300 amplitude and latency depend stronger on other factors besides stimulus modality, such as stimulus discriminability, intensity and probability [58], [59], [60], [61]. This is in accordance with auditory P300 BCI studies using stimuli other than spoken words which can be discriminated easier and faster. In such studies auditory and visual P300 latencies have been found to be identical [62]. Thus, we assume the differences in latency were caused by differences in stimulus discriminability rather than modality.

### Correlation between auditory oddball ERPs and BCI performance

Figures 8 and 9 provide an overview which ERP components correlated with BCI performance. In both paradigms a late component between 400 to 600 ms correlated with performance. This spatiotemporal distribution fits the characteristics of an anterior-negative, posterior-positive slow wave [63]. In the visual P300 BCI a strong positive correlation with performance was found on frontal electrodes around 200 ms. The spatiotemporal characteristics of this component indicate this to be the N2 ERP component [64]. Finally, it is quite surprising that there were no strong correlations in the time range of 300–400 ms, in which the P300 ERP component would be expected to correlate with performance.

When correlating the auditory standard oddball data with the performance achieved with either the auditory or the visual P300 BCI the strongest differences between low and high aptitude users were found in a late ERP component that was more negative at frontal and more positive at occipital and parietal channels for high as compared to low aptitude users (see Figures 8 and 9). For the visual P300 BCI this difference was visible on the individual level. This ERP component constitutes a late positive potential or anterior-negative, posterior-positive slow wave [63]. An enhancement of anterior-negative, posterior-positive slow wave has been found in ERPs following tones that require a response (e.g. button press) compared to ERPs following tones that do not require a response, rendering them indicative of a higher state of attentiveness [65]. Slow waves following a warning signal have also been attributed to be a component of the orienting response [66]. Similar in morphology and distribution to the orienting response is the “reorienting negativity” which in contrast has been observed to be specific to deviant tones and may therefore be directly applicable to our results [67]. This orienting response occurred in a time segment of 400–600 ms after stimulus presentation and was frontocentrally distributed. Therefore the late ERP component that we found to discriminate high from low aptitude users may be an indication of successful allocation of attention to the necessary switches between deviant and standard tones.

Correlations between N2 amplitude and performance were also observed. The N2 is not merely a sensory component but is also involved in cognitive control processes such as response inhibition, response conflict and error monitoring [64]. The N2 can also be subdivided into N2a, N2b and as was proposed by [68] and [69] into N2c components. In contrast to the N2a, the N2b and N2c components require attention to the stimulus and are accompanied by the elicitation of a P3 component, thus indicating that the observed N2 component in this work belongs to the N2b or N2c category. According to [64] the N2b tends to be larger for non-targets whereas the N2c tends to be larger for targets. As can be seen in Figure 7 the N2 elicited by the target stimuli (continuous line) is larger, thus supporting the assumption that the observed component may be categorized as an N2c. [64] also state that particularly the frontocentral N2 in response to rare auditory targets is usually a mixture of MMN, N2b and N2c. Indicative of at least a contribution by the N2b to the observed N2 component is its association with the orienting response which was discussed [70].

Quite unexpectedly, the P300 did not correlate with performance. This may in part be due to the fact that our sample consisted of healthy participants. Only two out of forty were unable to control the BCI. Thus in this sample a binary predictor (predicting whether the user is completely unable to control the BCI or has the potential for at least minimal control of the BCI) of P300 BCI performance cannot be evaluated. The P300 ERP component may very well be such a predictor. Possibly the P300 itself may be a better predictor of binary BCI performance than N2 or late potentials in a sample that includes patients that are completely unable to control a P300 BCI. Using the P300 ERP component for prediction of performance in a sample of BCI users with at least minimal potential to control a BCI may be further confounded by the knowledge that not only the P300 contributes to BCI performance. Usually time segments from 0 to 800 ms after stimulus presentation and electrodes on several scalp locations are used to record the EEG and extract features. Some publications have addressed the fact that the so-called P300 BCI is more of a general ERP BCI (see e.g. [71]). In fact the authors report that about 30% of all BCI users control the P300 BCI using a negative component around 200 ms. In our sample the N2, probably as a general indicator of attention, was a better predictor than the presence or absence of a P300. This is very plausible due to the fact the the sample consisted of healthy individuals with no known neurological disorders. We will show in a future study with ALS patients to what extend this finding is applicable to a patient population.

### Practical application of aptitude prediction

Two approaches to a future screening protocol to determine P300 aptitude are possible. On an individual level the findings from this paper, i.e. which ERP components predict aptitude, can be employed. Once larger datasets of users with known aptitude become available we would suggest using a data-driven approach to train classifiers that can predict the performance of future users based on the existing data. This is the approach we intend to implement for BCI end-users. The advantage is that a subjective assessment of the data to predict aptitude will not be required. One of the disadvantages is that the data needs to be recorded in an identical manner for each user and that the aforementioned large database needs to be collected.

The most probable procedure of employing aptitude prediction methods (a method to describe motor imagery BCI aptitude was described in [29] and other predictors of P300 BCI performance such as [36]) would be to assess the patients aptitude for all available BCI paradigms, say motor imagery and P300 BCIs, and then proceed to train and acquaint the patient to the paradigm for which he or she displays the highest level of aptitude. For example the aptitude prediction would be the basis for an informed decision about which BCI paradigm to apply. This would reduce frustration and strain on the patient and his surroundings.

## Conclusions

Correlations between individual values of the channel by time data matrix of the auditory oddball response and BCI performance were as high as 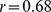. Correlations between individual components, e.g the N2 on Cz, were as high as 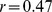. This proves the strong relationship between auditory oddball ERPs and subsequent BCI performance. Lack of correlation between the P3 component of the auditory oddball response and BCI performance supports the observation that P300 BCIs are not solely controlled by the P3 component but by a variety of ERP components elicited by the visual or auditory stimuli. Before practical use, it has to be evaluated whether the results transfer to patients. When attempting to communicate with non-responsive patients a fast screening method is of particular interest to quickly determine the most promising BCI.
